# Measuring inequalities in the distribution of the Fiji Health Workforce

**DOI:** 10.1186/s12939-017-0575-1

**Published:** 2017-06-30

**Authors:** Virginia Wiseman, Mylene Lagarde, Neha Batura, Sophia Lin, Wayne Irava, Graham Roberts

**Affiliations:** 1 Department of Global Health and Development, London School of Hygiene & Tropical Medicine, London, WC1E 7HT UK; 2 School of Public Health and Community Medicine, University of New South Wales, Kensington, NSW 2033 Australia; 3 London School of Economics and Political Science, Houghton Street, London, WC2A 2AE UK; 4 Institute for Global Health, University College London, Gower St, Kings Cross, London, WC1E 6BT UK; 5 Centre for Health Information Policy & Systems Research, College of Medicine Nursing and Health Sciences, Fiji National University, Suva, Fiji; 6 Human Resources for Development Alliance, PO Box 10570, Laucala Beach Suva, Fiji

**Keywords:** Health workers, Equity, Human resources for health, Distributional inequalities

## Abstract

**Background:**

Despite the centrality of health personnel to the health of the population, the planning, production and management of human resources for health remains underdeveloped in many low- and middle-income countries (LMICs). In addition to the general shortage of health workers, there are significant inequalities in the distribution of health workers within LMICs. This is especially true for countries like Fiji, which face major challenges in distributing its health workforce across many inhabited islands.

**Methods:**

In this study, we describe and measure health worker distributional inequalities in Fiji, using data from the 2007 Population Census, and Ministry of Health records of crude death rates and health workforce personnel. We adopt methods from the economics literature including the Lorenz Curve/Gini Coefficient and Theil Index to measure the extent and drivers of inequality in the distribution of health workers at the sub-national level in Fiji for three categories of health workers: doctors, nurses, and all health workers (doctors, nurses, dentists and health support staff). Population size and crude death rates are used as proxies for health care needs.

**Results:**

There are greater inequalities in the densities of health workers at the provincial level, compared to the divisional level in Fiji – six of the 15 provinces fall short of the recommended threshold of 2.3 health workers per 1,000 people. The estimated decile ratios, Gini co-efficient and Thiel index point to inequalities at the provincial level in Fiji, mainly with respect to the distribution of doctors; however these inequalities are relatively small.

**Conclusion:**

While populations with lower mortality tend to have a slightly greater share of health workers, the overall distribution of health workers on the basis of need is more equitable in Fiji than for many other LMICs. The overall shortage of health workers could be addressed by creating new cadres of health workers; employing increasing numbers of foreign doctors, including specialists; and increasing funding for health worker training, as already demonstrated by the Fiji government. Close monitoring of the equitable distribution of additional health workers in the future is critical.

## Background

All aspects of a health care system ultimately depend on people to run effectively. Evidence shows that the prospects for achieving desirable levels of coverage (80%) of measles immunization and skilled attendants at birth are greatly enhanced where health worker density exceeds 2.3 per 1,000 population [1]. Despite the obvious centrality of health personnel, the planning, production and management of human resources for health (HRH) remains the least developed aspect of health systems policy and development in many low and middle income countries (LMICs) [2]. In 2006, the World Health Organization (WHO) identified a global shortage of 2.4 million doctors, nurses and midwives [3]. It was estimated that countries which fall below a threshold of 2.3 health workers per 1000 population will struggle to attain 80% coverage in skill birth attendance and childhood vaccination [3]. This density ratio is therefore not so much a measure of *sufficiency* but rather represents the minimum requirement for achieving 80% coverage [3]. The estimated health worker density ratios for Fiji in 2009 consist of only 0.4 practicing physicians and 2.2 nurses and midwives per 1000 people [4].

While countries grapple with policies to address national shortages of health workers, many are asking what can be done in the interim to adjust the spread of existing health workers to better address growing health care needs. In the Pacific region, doctors are generally employed in hospitals in urban areas, while nurses deliver the majority of health services in rural areas [5]. In Fiji, there is inconsistency in size and population catchment for similar levels of health facility, with some facilities with small workloads being better equipped and staffed than others with much larger workloads [6].

Policies designed to address the maldistribution of health workers must be informed by robust analyses of not only the current per capita distribution of health workers but just as importantly, the distribution according to the level of ‘need’ within a country. The equitable distribution of health workers may not necessarily translate into an equal number of health workers across states, provinces or divisions. Just as it is argued at the global level that the number of health workers should reflect relative need, so is the case at the sub-national level where significant differences in morbidity and mortality patterns exist within countries [7]. For example, a recent study measuring the distribution of health workers at the sub-national level in Brazil found that the poorest and neediest states of Brazil experience the highest shortage of health workers and at the same time have the highest inequalities in the distribution of skilled health workers [8]. Similar findings have been reported for other LMICs including Vietnam [9], China [10], India [10] and Tanzania [7] where nurses and physicians are typically shown to be concentrated in wealthier urban areas of these countries.

Until now Pacific countries like Fiji have rarely been the subject of these types of analyses that have instead been limited to a small set of studies with access to reliable disaggregated data routinely collected at the country level. Moreover, there have been important advances in the methods used to measure inequalities in other fields of research that have not always been taken advantage of by those analysing inequalities in HRH [8]. Consequently, debates about appropriate policy solutions and workforce strategies have tended to proceed in countries like Fiji without good evidence on distributional inequalities in the health workforce at the sub national level. This paper seeks to measure inequalities in the distribution of the existing stock of health workers in Fiji and to account for the sources of these inequalities. From a methodological point of view, this paper not only contributes to the HRH literature by applying techniques traditionally confined to the measurement of income inequalities in economics, but it also examines how different measures of health care ‘need’ influence the allocation of health care resources, in this case, health workers. Finally, most studies focus on the distribution of a single cadre of health worker [11–13]. In this study we describe the distribution of all the main cadre of health workers and in this way, we are able to provide insight, for instance, into whether areas that have relatively few physicians are “compensated” by having relatively more lower-cadre workers.

## Methods

### Setting

Fiji is a middle-income country with a reported gross national income per capita of USD$4,830 in 2015 [14]. It has an estimated 2010 population of 854 000 [15] and comprises 332 islands and coral atolls, about 110 of them inhabited and covering more than 18,000 square kilometres. For government planning purposes the population is divided into four divisions^1^ and 15 provinces [16]. The nature of this geography poses significant challenges for the delivery of health services to the population that are dispersed over such a large maritime region [17]. The Government of Fiji finances and provides the majority of health services. Around 70% of health workers are salaried staff of the Ministry of Health (MoH). Nurses represent almost two-thirds of the health workforce.

Infant mortality rates in Fiji have declined from 60 deaths per 1000 live births in 1945 to around 20 per 1000 live births in 2000, where it has since remained stable [18]. Maternal mortality rates have been halved since the 1960s [18] but at a ratio of 29.3 per 100,000 live births, it is still well above the 2015 Millennium Development Goals (MDG) target of 10.3 per 100,000 live births [19]. The under-five mortality rate, which has barely changed since 2000, remains at 16.6 per 1000 live births, against the MDG4 target of 5.5 [19]. Despite improvements in infant and maternal mortality since the mid-20^th^ Century, the life expectancy of Fijians has not improved since 1985, and has fallen in indigenous Melanesian women, largely due to increases in premature mortality from non-communicable diseases (NCDs) [20]. NCDs are the leading cause of ill-health, disabilities and death in Fiji with around 80% of all deaths caused by an NCD [21]. Amongst NCDs, cardiovascular disease (CVD) is the main cause of death [18]. High prevalence and incidence of CVD risk factors such as hypertension [22], tobacco smoking [23], obesity and type 2 diabetes [24, 25] are attributed to poor lifestyle choices including high density, low nutrient diets and insufficient physical activity. Fiji currently faces a triple burden of diseases (communicable diseases, NCD, and injuries) common to a growing number of LMICs.

It is yet to be seen how Fiji will fit into the new 2030 agenda of the Sustainable Development Goals (SDGs), but with NCDs already at epidemic levels, the health system faces some serious challenges especially in the context of HRH [4]. Staff shortages and the need to strengthen the health system through improving investment in the health workforce has been identified as a key factor undermining progress towards the health related MDGs and SDGs [26]. Staff shortages are fuelled by two separate but compounded factors. On the one hand, there is increasing pressure to create a health workforce in Fiji to improve efficiency and resource allocation [17]. On the other hand, the Ministry of Health struggles to retain staff due to the international migration of skilled health workers, the internal migration of health workers from rural to urban areas, and from the clinical sector to the non-clinical sector [27].

### Data

The main data sources for this analysis were: the 2007 Population Census [28]; Ministry of Health records of crude all-cause death rates across divisions and provinces; and Ministry of Health personnel records that include data on age, sex, employment number, qualifications, place of work, position title, specialisation and location of health personnel. Cadres of health workers were grouped according to the International Labour Organisation’s International Standard Classification of Occupations (ISCO-08) [29].

### Measures of inequality

First, for each of the three categories of health workers, we calculated densities per 1000 population across the four divisions and 15 provinces. Then, to characterise inequalities in health workforce distribution, we calculated the following inequality indices: decile dispersion ratios; Lorenz Curve and Gini Coefficient; and the Theil index. The decile dispersion ratio measures the “distance” between two groups located close to the extremes of the distribution of a particular resource. This ratio can be calculated for a range of different percentiles but in general is used with the 10^th^ and 90^th^ percentiles [30]. For example, if the average number of health workers per 1000 population at the 10^th^ percentile is 2, and the average number of doctors per 1000 population of the 90^th^ percentile is 20, the ratio will be equal to 10. This measure is relatively robust to the existence of outliers in the distribution but it uses only two points of the entire distribution, thereby ignoring a lot of information.

The Gini Coefficient is a measure of the aggregate level of inequality and varies between 0, which reflects complete equality and 1, which indicates complete inequality (one person has all the income or consumption, all others have none). The Gini coefficient is based on the Lorenz curve, a cumulative frequency curve that compares the distribution of a specific variable (in this case, the number of health workers per province or division) with the uniform distribution (i.e. total population per province or division) that represents equality [31]. Compared to a decile dispersion index, the Gini coefficient incorporates all data and allows direct comparison between units with different population size.

The Theil index is another measure of inequality that permits sub-groups to be broken down or ‘decomposed’ within the context of larger groups [32]. The Theil Index involves more than a simple difference or ratio, they enable units (e.g. countries) to be partitioned into mutually exclusive and exhaustive groups (in this case, divisions and provinces) and two separate components of overall inequality to be calculated: a weighted sum of ‘within group inequality’ and a ‘between group’ component that measures inequality due solely to variations in health worker density across groups [10]. A Theil Index of 0 represents perfect equality and something close to 1 is considered very unequal. For this study, the Thiel L index is used to analyse between division and province inequalities – its formulation and decomposition properties are explained in detail elsewhere (see [10, 32]).

Finally, we introduce a measure of ‘need’ to explore further issues of health workforce inequalities. In health workforce planning, one typically compares the number of health workers per capita across geographical or administrative units [12, 33]. This is in essence what is done by the first three indicators. This implicitly assumes that the need for health workforce is fully correlated with population size. While this approach is the norm in many LMICs, mostly because routinely available data can be very limited, population levels may not be a very good measure of health care needs. Disease patterns and access to health facilities can vary significantly between divisions or provinces; areas with a smaller population may suffer from a larger share of disease burden. Also, a higher number of staff per capita might be needed in areas with a lower population density [7]. Consequently, analysts have started to incorporate alternative measures of need in the analysis of health workforce distribution, including Infant Mortality Ratios, Standardised Mortality Ratios, Under Five Mortality Ratios and HIV prevalence rate. [7, 8]. In the absence of Standardised Mortality Rates at the provincial level, here we use crude all-cause death rates as a proxy for health care needs.

All statistical computations were conducted in Stata version 14.

## Results

Table 1 shows the descriptive statistics of health workforce distribution, with the total number and densities of nurses, doctors and all health workers at the provincial and divisional level. Eastern division, which consist mainly of the remote islands, has far fewer nurses and doctors compared to all other divisions. However, looking at health worker density per population is more helpful as this captures the particular geography of Fiji. This shows that the ratio for health workers tends to be around the absolute minimum standard set by the WHO of 228 health workers for every 100,000 people (i.e. equivalent to a density ratio of 2.3 doctors, nurses and midwives per 1,000 population). Of the 15 provinces in Fiji, nine are below the minimum ratio for nurses and all are below the ratio for doctors. Of the four divisions, one is below the minimum ratio for nurses and all are below the ratio for doctors.

**Table 1:** Numbers and densities of health workers, 2011

Province	Numbers			Density (per 1,000 population)		
	Doctors	Nurses	All health workers	Doctors	Nurses	All health workers
Central division	179	925	1,262	0.50	2.56	3.50
Naitasiri	9	61	77	0.05	0.35	0.44
Rewa	152	742	1102	1.51	7.39	10.98
Serua	5	30	41	0.26	1.55	2.12
Namosi	0	7	7	0	0.95	0.95
Tailevu	13	85	117	0.22	1.45	2.00
Eastern division	17	123	149	0.44	3.16	3.83
Kadavu	2	28	33	0.19	2.69	3.17
Lau	13	76	95	1.28	7.47	9.33
Lomaiviti	1	9	12	0.06	0.54	0.73
Rotuma	1	10	13	0.57	5.67	7.37
Northern division	89	372	509	0.66	2.75	3.77
Bua	5	34	44	0.36	2.45	3.17
Cakaudrove	12	103	131	0.23	2.01	2.55
Macuata	72	235	342	1.03	3.36	4.9
Western division	151	657	895	0.46	2.00	2.73
Ba	144	528	780	0.60	2.21	3.26
Nadroga-Navosa	5	71	88	0.08	1.18	1.47
Ra	2	58	67	0.07	2.00	2.31

The absolute number of doctors is strikingly low in 9 out of 15 provinces where there are fewer than 10 doctors in post. In fact, the vast majority of doctors work in three provinces (Rewa in Central division, Macuata in Northern division and Ba in Western division), which include the capital (Suva). This is a reflection of the location of Fiji’s major hospitals. Admissions to these hospitals are from further afield than the provinces in which they are located. However, it is again important to balance this against population size - for example, in Kadavu province there are 0.19 doctors for 1,000 people, even though there are only 2 doctors in that province. With the possible exception of Rewa, Kadavu, Lau and Macuata, there is no clear indication at the provincial level that the shortage of doctors is being compensated through additional nurses. Health worker densities fall below the WHO norm for 6 of the 15 provinces.

Table 2 shows the three summary statistics of the inequality in the distribution of health workers in Fiji: decile dispersion ratios, Gini coefficient and Theil’s L index. Several observations about these estimates can be made. First, the overall inequality in the distribution of the health workforce is much higher when calculated at the lower level (provinces) compared to higher level (division), for all categories of health workers. For example, across provinces, a Gini coefficient of 0.532 for doctors, 0.412 for nurses and 0.434 for all health workers. Secondly, there is consistently higher inequality in absolute terms for doctors than all other categories – overall, within province and between province and for all three indices.

**Table 2:** Measures of inequality in health worker distribution across divisions and provinces

	Measures across divisions			Measures across provinces		
	p90/p10	Gini coefficient	Thiel’s L index	p90/p10	Gini coefficient	Thiel’s L Index
Nurses	1.52	0.077	0.011	13.57	0.412	0.513
Doctors	1.51	0.088	0.013	21.11	0.532	1.038
All health workers	1.39	0.059	0.008	15.91	0.434	0.581

The greater inequalities in health worker densities at the provincial level is confirmed by the decile dispersion ratios which express those populations with greatest availability of health workers as multiples of those that have least availability. For example, Table 2 shows that those that have greatest access to doctors (90^th^ decile) have about 21 times the number of nurses per 1000 than those in 10^th^ decile at the provincial level. This is compared to about 1.5 times at the divisional level.

Table 3 shows the decomposition of health workforce inequalities based on the Thiel L Index. Overall inequality in the distribution of the health workforce between provinces is much higher compared to overall inequality between divisions for all categories of health workers. For example, across provinces, a Thiel L index of Gini coefficient of 0.441 was reported for doctors, 0.278 for nurses and 0.302 for all health workers.

**Table 3:** Decomposition of Theil L indices, for divisions and provinces

	Between-division inequalities	Between-province inequalities
Doctors	0.012	0.441
Nurses	0.011	0.278
All health workers	0.008	0.302

**Fig. 1:**
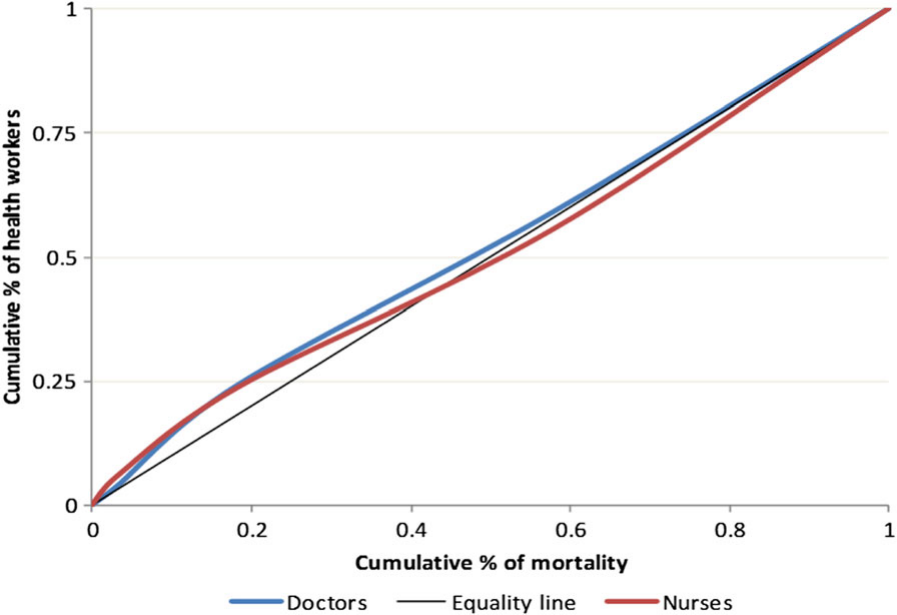
Lorenz curve showing the distribution of health workers according to level of need (mortality) at the Divisional Level

The Lorenz Curve in Fig. 1 shows the cumulative share of health workers against the cumulative share of need/mortality when divisions are ranked from those in lowest need (i.e. lower number of deaths) to highest need. The diagonal line represents a perfectly equal distribution of health workers (i.e. those with lower level of need, say 20%, would receive only 20% of health workers). Unequal distributions have a curve and the nearer the curve to the diagonal, the greater the degree of equality. Figure 1 shows that at the divisional level the share of health workers increases almost in proportion with need (Gini Coefficient = 0.059).

**Fig. 2:**
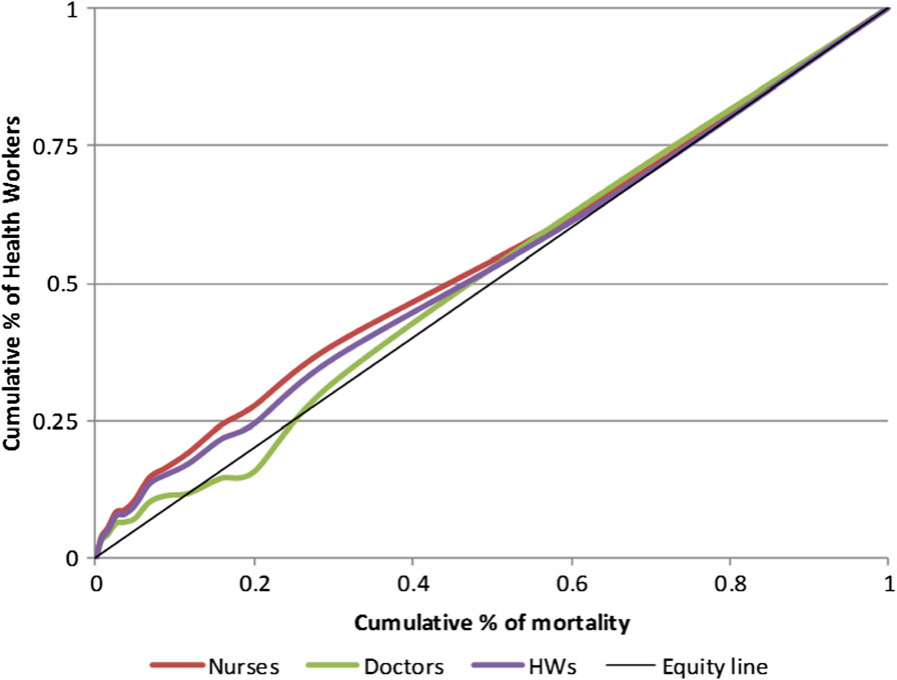
Lorenz curve showing the distribution of health workers according to level of need (mortality) at the Provincial Level

At the provincial level (Fig. 2) the Lorenz curves for all categories of health workers remain quite flat but there are some slight inequalities (Gini Coefficient = 0.434). For example, those better off (meaning they have lower mortality) tend to have a slightly greater share of health workers than they would if the distribution were perfectly equitable. Moreover, for doctors there seems to be a few provinces where mortality is low that receive a lower share of doctors than what they would be expected to receive if things were totally equitable.

## Discussion

HRH plays a pivotal role in strengthening health systems and in achieving the Sustainable Development Goals (SDGs) [34]. Findings from this study highlight the significant shortages of health workers that exist in several provinces of Fiji – six provinces fall short of the recommended threshold of 2.3 health workers per 1,000 people. The Fiji Government has been taking steps to address the shortage; for example, by creating new cadres of health workers, including nurse practitioners; employing increasing numbers of foreign doctors, including specialists; and increasing funding to the Fiji National University to increase the number of students to be trained as nurses and doctors [27].

Many other LMICs including countries such as China and India with the largest and most diverse health labour markets, have also implemented significant HRH reforms in the past decade. But despite improvements in the growth of their workforce, they continue to have significant HRH challenges, such as having sufficient numbers of qualified health workers who are equitably distributed geographically to meet local health needs [35]. There is an urgent need for HRH research and planning based upon the health needs of people and the skills and knowledge required to meet those needs. Simply increasing the numbers of health workers has not addressed the systemic challenges; universal health coverage and the SDGs require a discourse going beyond HRH shortages [36].

Governments like that of Fiji are therefore being forced to think more carefully about current allocations of health workers at the sub national level. This study systematically measured the level of inequality in the distribution of the existing health workforce in Fiji. Three different measures of inequality were used, decile dispersion ratios, the Gini Coefficient and the Theil L Index. Together, these measures form a consistent picture that while inequalities exist at the provincial level in Fiji, mainly with respect to the distribution of doctors, these inequalities are relatively small. Using a measure of need defined in terms of crude deaths, the data shows that health workers tend to be located in areas where need is greatest. This suggests that the Fijian Government is responding to health care needs as best it can using its available stock of health workers and that it must focus its efforts on policies to increase national shortages, most notably of doctors and specialists.

Two methodological issues are noteworthy. Currently the major criterion for allocating health workers across divisions in Fiji and many other countries in the region is relative population levels. While the current allocation of health workers based on relative population levels appears to align quite well with an allocation based on the crude death rate, caution is needed. For those countries where disease burden is distributed fairly evenly, then population size is probably the simplest criterion for allocating health workers. However, disease burden can change rapidly and those living in poverty are more susceptible to many diseases including HIV/Aids and tuberculosis. In these contexts, resource allocation formula based on population size alone is likely to lead to inequalities in the distribution of health workers. Analyses of the distribution of different types of health workers need to be regularly undertaken especially when new reforms such as the intake of foreign doctors and specialists or new cadres of health workers are introduced. The results from this analysis would serve as a useful baseline against which to measure the impact of these very recent HRH initiatives in Fiji.

Second, in the absence of alternative comprehensive measures of health care needs, this study relied on the total number of deaths per 1000 people to measure health care needs. Ideally, to compare mortality rates across different population groups or time periods, the rates should be “standardized "to a population with the same age structure. This data was not available at the provincial level in Fiji. While not a major concern for Fiji, variability in the accuracy of all-cause mortality data has also been highlighted as a problem for some other LMICs [37]. There would also be value in repeating this analysis using alternative measures of need. While crude deaths may be a good proxy for health care needs in areas with high death rates (indicative of an ageing population which requires labour-intensive health services), they are less suitable in low-income countries where around a third of annual deaths in children under the age of 5 years resulting from illnesses that can be prevented by interventions delivered through the health system [38] [7]. Again, age standardised mortality rates were not routinely available for provinces at the time of this study but these data gaps may be overcome through subsequent Demographic and Health Surveys.

This study uses SMR as a measure of need but considering the epidemiologic transition from communicable to NCDs, simply looking at doctors and nurses is probably too crude. With the growing burden of NCDs in the Pacific, a range of support staff including nurses, podiatrists, nutritionists and other allied health workers to support doctors would be required.

## Conclusions

This study, which borrows methods from the economic literature to explore inequalities in health workforce distribution in Fiji, illustrates the value in looking beyond the aggregate or national level when conducting these types of analyses. In particular, decomposition indices of inequality can be extremely useful when trying to separate out inequalities between groups and within groups. In this study, within division inequality accounted for the vast majority of inequality observed in the distribution of all categories of health workers. While the better off (those with lower mortality) tended to have a slightly greater share of health workers, the overall distribution of health workers on the basis of need was shown to be relatively equitable in Fiji. Countries should not see these types of analyses as one-off exercises, we strongly advocate they be repeated as data sources improve (both in terms of health worker numbers and disease burden) and as a means of evaluating significant HRH reforms.

## Abbreviations

CVD: Cardiovascular Disease
HRH: Human Resources for Health
ISCO: International Standard Classification of Occupations
LMICs: Low and Middle Income Countries
MDGs: Millennium Development Goals
MoH: Ministry of Health
NCDs: Non-Communicable Diseases
SDGs: Sustainable Development Goals
USD: United States Dollar
WHO: World Health Organization

## Footnotes

1 Based on the 2007 population census: Central ‐ with a population of 342,477, Eastern ‐ with a population of 39,313, Northern ‐ with a population of 135,961 and Western ‐ with a population of 319,611.
